# Trajectories of Functional Health in Later Life: The Role of Hearing Problems and Social Network

**DOI:** 10.1093/geroni/igaf122.4033

**Published:** 2025-12-31

**Authors:** Markus Wettstein, Bettina Williger, Lisa Incerti, Rosa-Linde Fischer, Julia Wolff, Susanne Wurm

**Affiliations:** Institute for Community Medicine, University Medicine Greifswald, Greifswald, Mecklenburg-Vorpommern, Germany; Engineering Psychology / Interdisciplinary Studies, Landshut, Bayern, Germany; Landshut University of Applied Sciences, Landshut, Bayern, Germany; ORCA Labs Europe, Erlangen, Bayern, Germany; IGES Institute, Berlin, Berlin, Germany; Institute for Community Medicine, University Medicine Greifswald, Greifswald, Mecklenburg-Vorpommern, Germany

## Abstract

Problems with hearing in midlife and old age are associated with poorer functional health. At the same time, the impact of hearing impairment on health outcomes varies considerably across individuals. Little is known about psychosocial factors that moderate the association between hearing impairment and functional health. We used data from the German Ageing Survey (2008-2023; n = 12,156; mean age = 62.35 years, age range 40-93 years) and investigated whether social network size is such a moderating factor, as a large social network might, for instance, provide support and motivation to maintain one’s functional health, particularly in older adults. Based on longitudinal multilevel regression models, controlling for age, gender, education, hearing aid use, self-rated health and region of residence, we found that functional health declined over time and was poorer at baseline in individuals with more self-reported hearing problems. A larger social network size was associated with better functional health at baseline, and among chronologically older adults, this association was stronger. In addition, this association was stronger in older adults who reported more hearing problems. A larger network size was also associated with a less steep functional health decline over time, but only among chronologically older adults. Our findings suggest that older adults, particularly those affected by hearing problems, may benefit from a larger social network which may help to counteract the detrimental effect of poor hearing on functional health. Interventions to increase social embeddedness in older adults, with and without impaired hearing, could thus contribute to promoting healthy aging.

